# The role of common genetic variation in educational attainment and income: evidence from the National Child Development Study

**DOI:** 10.1038/srep16509

**Published:** 2015-11-12

**Authors:** Neil M. Davies, Gibran Hemani, Nic J. Timpson, Frank Windmeijer, George Davey Smith

**Affiliations:** 1 Medical Research Council Integrative Epidemiology Unit, University of Bristol, BS8 2BN, United Kingdom; 2 School of Social and Community Medicine, University of Bristol, Barley House, Oakfield Grove, Bristol, BS8 2BN, United Kingdom; 3 Department of Economics, University of Bristol, 8 Woodland Road, Bristol BS8 1TN, United Kingdom

## Abstract

We investigated the role of common genetic variation in educational attainment and household income. We used data from 5,458 participants of the National Child Development Study to estimate: 1) the associations of rs9320913, rs11584700 and rs4851266 and socioeconomic position and educational phenotypes; and 2) the univariate chip-heritability of each phenotype, and the genetic correlation between each phenotype and educational attainment at age 16. The three SNPs were associated with most measures of educational attainment. Common genetic variation contributed to 6 of 14 socioeconomic background phenotypes, and 17 of 29 educational phenotypes. We found evidence of genetic correlations between educational attainment at age 16 and 4 of 14 social background and 8 of 28 educational phenotypes. This suggests common genetic variation contributes both to differences in educational attainment and its relationship with other phenotypes. However, we remain cautious that cryptic population structure, assortative mating, and dynastic effects may influence these associations.

Young people’s human capital accumulates over their childhood, partially via formal schooling, and is affected by their decisions and opportunities^1^,^2^. However, twin and family studies suggest that socio-economic characteristics, such as educational attainment, are heritable^3^. Branigan *et al.* (2013) meta-analysed twin studies from around the world that suggested a heritability of educational attainment due to additive genetic variation of 40.0% (95% confidence interval (95%CI): 35.3%, 44.7%). This implies genetics could play an important role in influencing educational attainment and could potentially explain some of the observed relationships between peoples’ backgrounds, education, and outcomes.

Recent studies have identified single-nucleotide polymorphisms (SNPs) associated with educational attainment and cognition^4^,^5^,^6^. Rietveld *et al.* identified three SNPs that were consistently associated with educational attainment. These were rs9320913, which was associated with the number of years of education, and rs11584700 and rs4851266, which were associated with graduating from college (university). Henceforth, we use “allele” or “alleles” to refer to the alleles that associated with higher educational attainment.

An alternative source of evidence about the relationships between genetic variation and educational phenotypes comes from estimates of the combined contribution of common genetic variation measured on genotyping arrays to these phenotypes using genome-wide data from unrelated individuals^7^. Henceforth, we refer to these estimates as “chip-heritability” to distinguish these estimates from the heritability studies using family data that account for all genetic variation (including rare variants). Marioni *et al.* (2014) found educational attainment had a chip-heritability of 21%, and socioeconomic position had a chip-heritability of 18%^8^. They also reported that education and socioeconomic position had a bivariate chip-heritability of 41%. This is the proportion of the phenotypic correlation between the two phenotypes that can be explained by shared additive SNP effects. For heritable traits a substantive bivariate heritability is a necessary (but not sufficient) condition for a causal relationship. Krapohl and Plomin (2015) found that common genetic variation explained 31% (SE = 0.11) of differences in educational attainment, and 20% (SE = 0.11) of the variation in socioeconomic position. They also found that education and socioeconomic position had a bivariate chip-heritability of 50%^9^.

There is still much that we do not know about the relationship between the genome and educational attainment and outcomes later in life, such as household income. Here, we provide new evidence from the National Child Development Study (NCDS) using three approaches^10^. First, we investigated the associations of the three SNPs described above on a range of educational and socio-economic phenotypes, and second we investigated the association of a genome-wide allele score constructed using all the coefficients reported in the educational attainment GWAS, and third we used genome-wide approaches to estimate the chip-heritability of educational attainment and the genetic correlation and bivariate chip-heritability of educational attainment and other phenotypes.

## Results

There were 17,416 infants that enrolled into the NCDS, of which 9,377 had biological data in the 2003 biomedical survey, 5,458 were genotyped and passed the quality control described in the methods below. See Supplementary Figure 1 for a flow chart of the participants’ inclusion and exclusion from the study, and Supplementary Table 1 for a description of their characteristics. Most participants (52.6%) reported having no O-levels (Supplementary Table 2). Of participants who reported having O-levels, the median was 4. A minority (19%) of participants stayed on for A-levels and 11% had been awarded a degree by age 23. The average household nominal income was £45,247, from all sources before taxes at age 46 in 2004, which is equivalent to £59,038 in June 2015 prices (Supplementary Table 2). This is comparable to figures from the UK Office of National Statistics which suggest that average household nominal incomes were £37,554 in 2004 for people aged 30 to 49^11^. The three SNPs were in Hardy Weinberg Equilibrium (Supplementary Table 3).

### Association of number of O-levels and phenotypes

Number of O-levels was associated with 42 of 44 observed phenotypes. Participants who achieved more O-levels had: older parents, taller mothers and fathers, more educated parents, fewer mothers who smoked, more fathers and grandfathers of high social class, and scored more highly in all measures of educational attainment at all ages (Tables 1, 2, 3, [Table t3]). Participants with more O-levels were more likely to obtain A-levels; to obtain a degree; and all measures of income (Table 4).

### Association of the 3 SNP allele score and the phenotypes

The 3 SNP allele score was associated with two measures taken from the perinatal survey: fathers’ and maternal grandfathers’ social class (odds-ratio = 1.07, 95%CI: 1.01, 1.14, p-value = 0.03 and odds-ratio = 1.08, 95%CI: 1.01, 1.15, p-value = 0.02, respectively) (Table 1). The score was also positively associated with paternal social class, however this association was small and imprecisely estimated (odds-ratio 1.01, 95%CI: 0.94, 1.09, p-value = 0.74). Adjusting for the first twenty principal components of the genotype data matrix did not meaningfully affect the results.

The 3 SNP allele score was positively associated with educational attainment at age 7 and 11 (Table 2). Each allele was associated with a higher likelihood of intending to stay on in school at age 11 (odds-ratio = 1.06, 95%CI: 1.00, 1.12, p-value = 0.03). The score was weakly associated with maths test score at age 16 (mean-difference = 0.12, 95%CI: −0.07, 0.30, p-value = 0.22), and was more strongly associated with reading test scores (mean-difference = 0.27, 95%CI: 0.11, 0.43, p-value = 0.001) (Table 3). There was little evidence that the 3 SNP allele score was associated with the teachers’ reports of the participants’ ability, or whether the participant was hardworking or lazy. It was positively associated with risk of poor eyesight (odds-ratio = 1.13, 95%CI: 1.03, 1.25, p-value = 0.01), but there was little evidence it was associated with having poor hearing, speech, or being clumsy. The 3 SNP allele score was associated with the participants’ aspirations for staying on past the school minimum age, studying A-levels and further full time study. These associations were not substantially affected by adjusting for the first twenty principal components, which suggests that population stratification is unlikely to explain our results.

**Figure 1:**
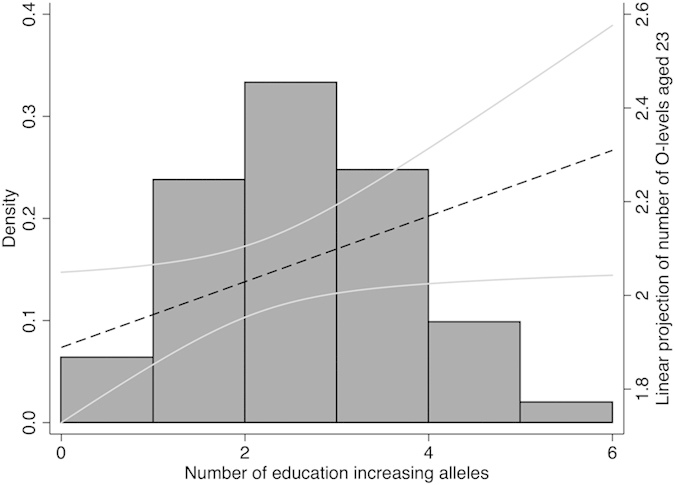
The association of the Rietveld *et al.* (2013) allele score and number of O-levels achieved at age 23. The dashed line indicates linear prediction of household income based on the number of alleles. The grey line indicates the confidence intervals of linear prediction.

**Figure 2:**
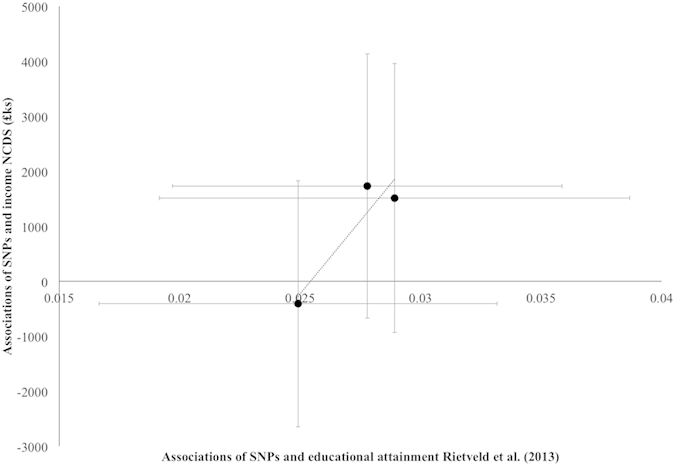
Associations of rs9320913, rs11584700, rs4851266 and household income at age 46 compared to associations with educational attainment reported by Rietveld *et al.* (2013). Point estimates and confidence intervals for the effects of the SNPs on education (x-axis) taken from Rietveld *et al.* Point estimates for the effects of the SNPs on household income (y-axis) taken from the National Child Development Study. The dashed line indicates the line of best fit between the points.

On average each extra allele was associated with an additional 0.07 (95%CI: 0.01, 0.14, p-value = 0.03) O-levels. Participants with no alleles achieved 1.70 O-levels, whereas those with five alleles achieved 2.30 O-levels (Fig. 1). Participants were 11.2% (95%CI: 4.7%, 18.2%, p-value < 0.001) more likely to achieve A-levels per allele (Table 4). Only 14.4% of 347 participants with no alleles had A-levels, whereas 25.4% of the 106 participants with 5 alleles had A-levels (Supplementary Figure 2). Participants were 3.6% (95%CI: −4.0%, 11.9%, p-value = 0.36) more likely to achieve a degree per allele. Finally, each additional education associated allele was associated with a 1.0% (95%CI: −1.3%, 3.2%, p-value = 0.41) higher household income (Table 4). The association of the three individual SNPs and educational attainment and before tax household income is shown in Fig. 2.

The association of the three SNP allele score on the number of O-levels was larger for children of high social class fathers compared to those of low social class fathers (Supplementary Table 4). However, we found little evidence of such an interaction for obtaining A-levels, obtaining a degree, or household income. Therefore the interaction of social class and number of O-levels may simply be due to chance.

Each extra O-level was associated with a 7.4% (95%CI: 6.5%, 8.3%, p-value < 0.001) higher before-tax household income. Participants with A-levels had 48.0% (95%CI: 41.7%, 54.2%, p-value < 0.001) higher household income than those without A-levels, and those with degrees had 55.7% (95%CI: 48.1%, 63.1%, p-value < 0.001) more than those without. The instrumental variable results implied a larger effect of education on earnings than the observational association, however the two-stage least squares results using the allele score were imprecise. The instrumental variable results using a saturated model and the continuously updating estimator were more precise and suggested that each additional O-level generated a 26.2% (95%CI: 1.3%, 51.1%, p-value = 0.04) increase in earnings, obtaining A-levels generated a 142.6% (95%CI: 5.8%, 279.3%, p-value = 0.04) increase in earnings and obtaining a degree generated a 524.3% (95%CI: −225.3%, 1274.9%, p-value = 0.17) increase in earnings compared with not having A-levels or a degree. The effect estimates are large and depend on strong assumptions that are unlikely to hold; see the discussion for further details.

The genome-wide allele score was positively associated with 41 of 47 traits (Supplementary Tables 5 to 10). The score was strongly associated with take home pay and savings and investment wealth: a one standard deviation increase in the score was associated with a 13.8% (95%CI: 10.0%, 17.6%) increase in net take home pay, and a 32.3% (95%CI: 22.5%, 42.1%) increase in wealth.

### Contribution of common genetic variation

Estimates of the chip-heritability for the social background and education related phenotypes are shown in Tables 5, 6, 7, 8. Overall we found evidence (p < 0.05) of chip-heritability for 6 of 14 background and 17 of 29 educational phenotypes. There was modest evidence of gene-environment correlations—in the form of non-zero estimates of chip-heritability for the background phenotypes, for example, the age the participants’ fathers and mothers left school (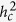 = 0.07 SE = 0.07 and 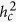 = 0.07 SE = 0.08, respectively Table 4). We found evidence of a moderate chip-heritability of mothers’ smoking behaviour during pregnancy (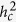 = 0.10, p-value = 0.04). We found positive genetic correlations between the participants’ father’s, and their paternal and maternal grandfathers’ social classes and the number of O-levels obtained (*r*_g_ = 0.87, p-value = 0.002, *r*_g_ = 0.39, p-value = 0.27 and *r*_g_ = 0.64, p-value = 0.03, respectively). There was substantial bivariate chip-heritability between the participants’ number of O-levels and their fathers’, and paternal and maternal grandfathers’ social classes (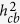 = 0.54, 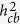 = 0.28, and 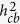 = 0.59 respectively).

There was moderate chip-heritability of the participants’ arithmetic and reading test scores, and their teacher reported ability at age 7 (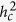 = 0.11 SE = 0.07, 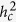 = 0.15 SE = 0.07, and 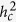 = 0.17 SE = 0.07 respectively) (Table 5), and for teacher reported ability there was a substantial genetic correlation between these phenotypes and number of O-levels achieved at age 16 (*r*_g_ = 1.00, p-value = 0.002). Maths and reading tests measured at age 11 were also moderately heritable (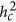 = 0.23, SE=0.07 and 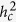 = 0.28, SE=0.07), as were the correlations between these phenotypes and the number of O-levels (*r*_g_ = 0.53 and *r*_g_ = 0.61 respectively Table 5). The heritability of maths and reading scores at age 16 were similar to at age 11 (Table 7). There was moderate heritability of parents’ wishes about their child’s education; the participants’ aspirations and intentions at age 16 about staying on in school to obtain A-levels and a degree; and of genetic correlations between these phenotypes and the number of O-levels. In contrast, there was little evidence from this sample that the participants’ speech, eyesight or clumsiness were due to common variation.

Finally, the heritabilities of the number of O-levels, obtaining A-levels or a degree were similar (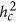 = 0.10, SE = 0.06, 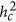 = 0.11, SE = 0.06 and 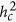 = 0.13 SE = 0.06 respectively, Table 8). There was evidence of large genetic correlations between obtaining A-levels and a degree and the number of O-levels. We found modest evidence that net take home pay, and wealth were heritable (Table 8).

## Discussion

In this study, we found that three SNPs: rs9320913, rs11584700, and rs4851266 were associated with educational attainment across childhood and years of education obtained by early adulthood. We found little evidence that the size of the genetic effects differed between reading and maths ability. The SNPs were also associated with 11-year-old children’s intentions to stay on in school after age 15 (the school minimum leaving age at the time of the study). Individuals with more alleles were more likely to obtain O-levels, A-levels and degrees.

Of the 29 measured education phenotypes 17 had detectable chip-heritability (p < 0.05). Common SNPs explained 10% of the variation in number of O-levels achieved and 13% of the reading scores at age 11, and 13% of the variance in obtaining a degree by age 23. Previous authors have reported evidence from twin studies suggesting that the heritability of educational phenotypes is relatively stable across childhood^12^. Consistent with this, we found little evidence that the contribution of common genetic variation changed over childhood and adolescence. However, our results are imprecise, reflecting relatively low statistical power.

Further, we found evidence of genetic correlations between the number of O-levels and 8 of 28 educational phenotypes. For example, the genetic correlations between the number of O-levels a participant achieved and their arithmetic at age 7 and Maths score at ages 11, and 16 were 0.50, 0.53, and 0.59 respectively. This suggests similar genetic pathways influence both the number of O-levels and students’ motivation as reported by the teachers. There were further genetic correlations between O-levels and parents’ interest in their children’s education at age 11. This suggests that a proportion of the associations of parents’ engagement with their children’s education and their children’s educational attainment may be due to a shared common genetic architecture.

Our genome-wide results are consistent with the results reported by Marioni *et al.* (2014)^8^. They found a bivariate heritability between cognition, educational attainment and socio-economic position of between 24% and 59%. Our point estimate of the bivariate chip-heritability of socioeconomic position and educational attainment is also consistent with results reported by Krapohl and Plomin (2015)^9^. Our results add to the growing evidence that socioeconomic gradients in educational attainment may be partially due to common genetic variation.

We also note that the putative positive bivariate chip-heritability between number of O-levels and parents’ and grandparents’ socio-economic position could be due to a number of factors, including pleiotropic effects, dynastic effects, assortative mating, and population stratification. The simplest explanation is a direct pleiotropic effect of common SNPs on both phenotypes, indicated by the arrow A on Supplementary Figure 4. However, it is also possible that there are dynastic (direct) effects of parents’ socioeconomic position on their offspring, as indicated by the B_1_ and B_2_ arrows on Supplementary Figure 4. Our analysis would attribute a portion of the direct effects of the parents’ socioeconomic economic position to common genetic variation. Finally, assortative mating between parents on socioeconomic position and educational attainment or hidden population stratification may lead to over-estimates of the contribution of common genetic variation to the association of socioeconomic position and educational attainment (arrows C_1_ and C_2_ in Supplementary Figure 4). Hence the estimated genetic correlation between these phenotypes is not conclusive proof that these phenotypes are due to a shared biological process.

A strength of our study is that we used a large geographically representative sample (N = 5,458). The SNPs were imputed using the 1,000 genomes reference panel. We found weak evidence that SNPs associated with higher educational attainment were also associated with higher adult household income. This is consistent with a causal effect of education on earnings. We also exploited extremely detailed data on participants’ educational attainment over childhood and adolescence. This provides important evidence about the aetiology of educational attainment and labour market success across the life-course.

The SNPs detected in Rietveld *et al.* are unlikely to be valid instrumental variables for educational choices at a specific point in time, such as “took A-levels”. This is because the three Rietveld *et al.* (2013) SNPs affect a range of phenotypes other than educational attainment. For example, we know that the SNPs are associated with cognition^6^. The SNPs also affect educational attainment and decisions across the life-course. Whereas the instrumental variable point estimates assume that the SNPs only affect earnings via the number of O-levels, having A-levels or having a degree. This would invalidate the use of these SNPs as genetic instrumental variables for educational attainment^13^. This could explain why our instrumental variable estimates of the effects of education on wages are so substantial. Population stratification could explain our results, however we excluded individuals who were not of European genetic origin and adjusting for the first twenty principal components did not affect the three SNP allele score results, so this is unlikely. We found that estimates of the contribution of common genetic variation dropped substantially when adjusting for principal components, and it may reduce further still if more accurate measures of cryptic population structure are used.

Common genetic variation explained more of the variability in children’s educational attainment than is typically attributed to teachers or schools^14^,^15^,^16^. These results add to a growing body of literature that suggests a portion of the observed differences in educational attainment between children can be explained by underlying genetic differences. Furthermore, the increasing availability of genome-wide datasets provides an opportunity to produce new evidence about the genome’s role in educational attainment and other important outcomes such as earnings. These findings are important as they suggest that commonly studied relationships, such as socioeconomic gradients in educational attainment may be substantially explained by common genetic variation. However, shared genetic variation could reflect genetic variation influencing one phenotype (e.g. education) which then influences outcomes, which would both generate genetic correlation. Advances in Mendelian randomization methods^17^, such as bidirectional Mendelian randomization, two-sample, and invalid (pleiotropic) instrument robust methods could potentially provide further insights into the development of educational phenotypes^18^,^19^,^20^,^21^.

## Materials and Methods

### The Data—The National Child Development Study (NCDS)

The NCDS is a nationally representative cohort study of 17,416 births in a single week in 1958. The participants and their families have been surveyed a further nine times during childhood, adolescence and as adults. We had information about the participants’ family’s socio-economic position at birth, their intermediate educational attainment at ages 7, 11 and 16 and their labour market outcomes, measured by their household income at age 46. For further details of the cohort see the published cohort profile^10^. We report the associations with a range phenotypes, for which the summary statistics can be seen in Supplementary Table 1.

### Perinatal Mortality Study 1958 shortly after birth

We defined the participants’ family background using the birth survey: including their mother’s and father’s age and height, mother’s weight, the age at which their parents left school, whether their mother smoked before and/or during pregnancy, and whether their fathers, and paternal and maternal grandfathers were in social class I or II.

### NCDS Survey 1 1965 at age 7

From this survey we used the participants’ academic ability, as reported both by arithmetic and reading tests and a summation of teacher reported Likert scores for awareness, ability at reading, creativity, number skills and speaking.

### NCDS Survey 2 1969 at age 11

From this survey we used the parents’ initiative to discuss the child’s education with the school and if the participants’ intended to stay on in school after the age of 15.

### NCDS Survey 3 1974 at age 16

We extracted the participants’ maths and reading test scores at age 16; the teachers’ opinion about whether the participant was above average ability in Math, English and Science; whether the participant was lazy or hardworking, which was measured on a Likert scale (1-5); teacher reported difficulties in hearing, speaking, eyesight and clumsiness; the parents’ positive attitude to their children’s education; whether the parents wanted the child to get a degree and expected them to stay in school after age 16; and finally whether the participant aspired to full-time study after leaving school.

### NCDS Survey 4 1981 educational attainment at age 23

We report three educational outcomes: 1) the number of school examinations (O-levels) each participant achieved at age 16; 2) whether the participant achieved A-levels at age 18; and 3) whether the participant completed a college (university) degree. Only a minority of students stayed on at school until age 18 to take A-levels, that are usually a requirement for attending university, (Supplementary Table 2). We used data from the survey at age 23, because this represents the educational achievement of the participants after they had passed through the conventional education system, but before their educational attainment could be affected by adult education.

### Income and wealth

We used four measures of income and wealth. First household income before taxes, second take home pay, third equivalised family income, and finally investment and savings wealth. Household income before taxes was measured in the NCDS Survey 7 2004 income at age 46. This includes labour market earnings, state and private pensions, state-benefits such as child benefits or tax credits, and investment income such as interest from savings and income from rental properties. Net take home pay is from the 2008 survey at age 50, and records income from labour market income only. Family equivalent net income is a derived variable from the 1981 survey at age 23. It measured the participants’ income accounting for family structure.

### Genome-wide data

The NCDS has biological samples from 9,377 of the participants who took part in a biomedical survey between 2002 and 2004. These samples were used to extract DNA for use in high-throughput genotyping arrays. These biological samples were originally genotyped in three different, but overlapping, samples. These results were used as control data for the first and second waves of the Wellcome Trust Case Control Consortiums^22^. Each sample had between 516,115 and 653,522 SNPs genotyped. Prior to imputation we excluded the SNPs with: a minor allele frequency of less than 1%, a Hardy Weinberg Equilibrium test p-value less than 1e-6. We also excluded individuals missing data for more than 3% of SNPs. We aligned SNP positions to the Human Genome 19 and flipped strands to be positive. We performed haplotyping using ShapeIt V2 software and imputed the data using Impute V2.2.2. The imputation used all population samples in the 1,000 genomes reference panel (phase 1 version 3, phased using ShapeIt v2 software, release 9-12-13)^23^,^24^,^25^. For whole genome variance estimation we retained HapMap3 SNPs with imputation quality score (R^2^) > 0.8 and a minor allele frequency of more than 1%^26^. This resulted in a final dataset of 1,187,090 individual SNPs. We excluded individuals not born in the UK for two reasons: they may have experienced different education systems, and they may introduce population stratification into our data. We combined the three genome-wide datasets to give a final sample of 5,458 individuals with genome-wide data.

We constructed an allele score equal to each participant’s number of G, T, and A alleles in rs11584700, rs4851266, and rs9320913 respectively. These SNPs were in strong linkage disequilibrium with genotyped SNPs, with IMPUTE 2 info-scores >0.99. In the Rietveld *et al.* GWAS these alleles were associated with higher educational attainment. We refer to this variable as the three SNP Rietveld allele score^27^. The NCDS was not included in the Rietveld *et al.* GWAS. We report the allele frequencies and tests for Hardy-Weinberg equilibrium in Supplementary Table 3. In a sensitivity analysis we repeated the allele score analysis using a weighted allele score of the three SNPs, these results were similar to the analysis using an unweighted score, so we do not discuss the weighted allele score results further.

### Statistical methods

We report the results of three sets of analyses: first, the associations of the Rietveld allele score and the background and educational phenotypes described above; second, the association of a genome-wide allele score and the phenotypes; and third, we report the chip-heritability, denoted 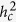, which is the proportion of each phenotype explained by common genetic variation measured on the genome-wide arrays^7^. This is a lower bound estimate of total heritability as estimated by twin studies, because chip-heritability does not account for the effects of a large proportion of rare variation or unmeasured common variation which is in linkage equilibrium with the measured SNP data.

We estimated the associations between the number of O-levels each participant achieved and their observable phenotypes listed in Supplementary Table 1 using linear and logistic regression for the continuous and binary phenotypes respectively. We compared these associations to the associations of each phenotype and the Rietveld allele score, again estimated using linear and logistic regression. We report mean differences for the continuous phenotypes and odds-ratios for the binary phenotypes. All variance estimates from linear models use sandwich estimators that allow for heteroskedasticity^28^. We also report the Rietveld allele score results adjusted for the first twenty principal components of the genotype data matrix to allow for population stratification. The principal components were estimated using PLINK^29^,^30^.

In an exploratory analysis, we investigated whether there was an interaction between socioeconomic position of the participants’ fathers and the effects of the allele score on educational attainment and household income. In a further exploratory analysis we investigated using instrumental variable analysis to estimate the effects of educational attainment on household income. We report instrumental variable estimates on household income of: the number of O-levels, whether they had A-levels, or had obtained a degree. We used two methods: first, two-stage least squares using the Rietveld allele score as an instrument and, second, a model in which each of the three genotypes was indicated by two binary variables representing how many alleles the participant had. This resulted in six binary variables. We were concerned that the latter model might suffer from weak instrument bias, therefore we report Newey-Windmeijer standard errors using the continuously updating estimator^31^,^32^,^33^. This estimator is robust to weak instruments and allows for a general form of heteroskedasticity.

We investigated the genetic architecture of educational attainment and socio-economic position using genomic restricted maximum likelihood (GREML) analysis in Genome-wide Complex Trait Analysis (GCTA) software^7^. GREML estimates the “chip-heritability” – the proportion of variation in a phenotype explained by common SNPs measured in genotyping arrays. We refer to this estimate as chip-heritability (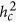,). For this analysis we inverse normal rank transformed the continuous variables. For each phenotype, we tested whether chip-heritability differed from zero using likelihood-ratio tests. We report the genetic correlation between the genome-wide genetic effects for O-levels and the other phenotypes, indicated by *r*_g_. We calculated the bivariate heritability of the number of O-levels and other phenotypes; we refer to this as the bivariate chip-heritability (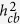). This parameter is the proportion of the correlation between number of O-levels and other phenotypes that can be explained by common genetic variation measured on genotyping arrays. We calculated bivariate chip-heritability using the following formulae 
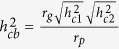
, where 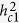 is the chip-heritability of the number of O-levels, 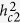 is the heritability of the second phenotype, and 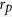 is the observed correlation between the two phenotypes. All estimates of chip-heritability adjust for indicator variables for the genotyping sample and the first twenty principle components of the genotype data matrix.

### Statistical power

To maximise the power of our study we only investigated the association of the three SNPs found to associate with educational attainment at genome-wide levels of significance (p < 5e-8) in Rietveld *et al.* (2013). This reduced the number of statistical tests we had to run and increase our power to detect associations. This approach was used in Rietveld *et al.* (2014) to investigate the association of these SNPs and cognitive ability.

There were 1,034 and 586 of the participants who obtained A-levels and degree. We used Visscher’s method to calculate the likely power of our chip-heritability estimators^34^. Thus, assuming a heritability of 0.3 and a variance of the genetic relatedness matrix of 2e-5, we had a 70% and 45% power to reject the null hypothesis that 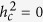. For continuous traits we had greater power, 93.67% and 99.95% assuming heritabilities of 0.2 and 0.3 respectively.

### Data access

The data can be accessed by submitting an application to the NCDS^35^. The statistical code used to create these results can be accessed here (https://github.com/nmdavies/NCDS).

## Additional Information

**How to cite this article**: Davies, N. M. *et al.* The role of common genetic variation in educational attainment and income: evidence from the National Child Development Study. *Sci. Rep.* **5**, 16509; doi: 10.1038/srep16509 (2015).

## Supplementary Material

Supplementary Materials

## Figures and Tables

**Table 1:** Association of number of O-levels and Rietveld *et al.* (2013) allele score and perinatal covariates.

Outcome:	N	Number of O-levels				Rietveld *et al*. (2013) allele score							
		Unadjusted				Unadjusted				Adjusted for population stratification			
			Confidence interval				Confidence interval				Confidence interval		
		Mean difference	Lower	Upper	P-value	Mean difference	Lower	Upper	P-value	Mean difference	Lower	Upper	P-value
Father’s age (years)	5,100	0.149	0.088	0.209	<0.001	−0.010	−0.163	0.144	0.90	−0.018	−0.172	0.136	0.82
Mother’s age (years)	5,255	0.145	0.092	0.197	<0.001	−0.048	−0.181	0.084	0.47	−0.054	−0.188	0.079	0.43
Mother’s weight (kg)	5,152	−0.017	−0.104	0.069	0.69	0.119	−0.104	0.342	0.30	0.133	−0.090	0.356	0.24
Father’s height (cm)	4,535	0.349	0.278	0.420	<0.001	−0.078	−0.264	0.109	0.41	−0.063	−0.250	0.124	0.51
Mother’s height (cm)	5,054	0.226	0.166	0.286	<0.001	0.128	−0.026	0.282	0.10	0.154	0.000	0.309	0.05
Age father left school (years)	5,078	0.215	0.188	0.242	<0.001	0.006	−0.044	0.057	0.80	0.004	−0.046	0.055	0.87
Age mother left school (years)	4,124	0.175	0.152	0.197	<0.001	−0.001	−0.043	0.040	0.96	−0.001	−0.043	0.041	0.96
Average age parent’s left school (years)	3,874	0.205	0.181	0.229	<0.001	−0.014	−0.058	0.029	0.52	−0.016	−0.060	0.028	0.47
Male*	5,458	0.987	0.968	1.005	0.15	0.999	0.953	1.047	0.96	1.001	0.955	1.050	0.95
Mother smoked*	5,245	0.915	0.897	0.934	<0.001	1.007	0.959	1.057	0.79	1.003	0.955	1.054	0.91
Mother smoked during pregnancy*	5,197	0.902	0.882	0.922	<0.001	0.991	0.942	1.044	0.74	0.988	0.938	1.041	0.65
Father high social class*	5,023	1.251	1.222	1.280	<0.001	1.068	1.005	1.136	0.03	1.071	1.006	1.139	0.03
Paternal grandfather high social class*	4,409	1.129	1.100	1.159	<0.001	1.012	0.943	1.087	0.74	1.013	0.943	1.088	0.72
Maternal grandfather high social class*	4,367	1.156	1.129	1.184	<0.001	1.078	1.010	1.151	0.02	1.074	1.006	1.147	0.03

^*^Odds-ratios reported. Robust standard errors. Adjusted analysis includes first twenty principal components of the genotype matrix.

**Table 2:** Association of number of O-levels and Rietveld allele score and educational phenotypes at age 7 and 11.

Outcome:	N	Number of O-levels				Rietveld *et al*. (2013) allele score							
		Unadjusted				Unadjusted				Adjusted for population stratification			
			Confidence interval				Confidence interval				Confidence interval		
		Mean difference	Lower	Upper	P- value	Mean difference	Lower	Upper	P-value	Mean difference	Lower	Upper	P-value
Tests at age 7:													
Arithmetic	4,932	0.301	0.279	0.323	<0.001	0.111	0.052	0.169	<0.001	0.108	0.049	0.166	<0.001
Reading	4,917	0.146	0.135	0.156	<0.001	0.038	0.008	0.069	0.01	0.035	0.005	0.066	0.02
Teacher reported ability	4,951	0.561	0.529	0.593	<0.001	0.111	0.025	0.197	0.01	0.104	0.017	0.190	0.02
Tests at age 11:													
Maths	4,770	1.901	1.817	1.985	<0.001	0.196	−0.056	0.448	0.13	0.191	−0.062	0.443	0.14
Reading	4,771	1.061	1.010	1.112	<0.001	0.168	0.019	0.318	0.03	0.169	0.020	0.319	0.03
Verbal	4,773	1.451	1.378	1.524	<0.001	0.152	−0.069	0.374	0.18	0.150	−0.072	0.373	0.19
Nonverbal	4,773	1.147	1.086	1.208	<0.001	0.067	−0.111	0.244	0.46	0.064	−0.114	0.243	0.48
Copying	4,760	0.106	0.091	0.120	<0.001	0.011	−0.025	0.048	0.54	0.014	−0.022	0.051	0.44
Teacher reported ability	4,719	0.529	0.504	0.555	<0.001	0.055	−0.017	0.126	0.13	0.054	−0.018	0.126	0.14
Child’s intentions and expectations													
Parents interested in child’s education*	4,693	1.166	1.139	1.194	<0.001	1.002	0.952	1.055	0.94	1.004	0.953	1.058	0.88
Intends to stay on past min. age*	4,683	1.163	1.138	1.188	<0.001	1.062	1.005	1.122	0.03	1.063	1.006	1.123	0.03

Notes: *Odds-ratios reported. Robust standard errors. Adjusted analysis includes first twenty principal components of the genotype matrix.

**Table 3 t3:** Association of number of O-levels and Rietveld allele score and educational phenotypes at age 16.

Outcome:	N	Number of O-levels				Rietveld *et al*. (2013) allele score							
		Unadjusted				Unadjusted				Adjusted for population stratification			
			Confidence interval				Confidence interval				Confidence interval		
		Odds- ratio	Lower	Upper	P-value	Odds- ratio	Lower	Upper	P-value	Odds-ratio	Lower	Upper	P-value
Tests:													
Maths*	4,235	1.464	1.405	1.522	<0.001	0.109	−0.076	0.293	0.25	0.115	−0.070	0.301	0.22
Reading*	4,253	1.080	1.032	1.128	<0.001	0.276	0.114	0.438	<0.001	0.267	0.105	0.430	0.001
Teacher reported ability													
Maths	4,139	1.263	1.231	1.295	<0.001	1.017	0.948	1.090	0.64	1.018	0.949	1.092	0.62
English	4,223	1.165	1.139	1.192	<0.001	1.023	0.962	1.087	0.47	1.026	0.964	1.091	0.42
Science	3,543	1.214	1.181	1.248	<0.001	1.004	0.929	1.085	0.93	1.003	0.927	1.086	0.94
Lazy 1-2-3-4-5 Hardworking*	4,308	0.151	0.139	0.162	<0.001	0.017	−0.014	0.048	0.28	0.020	−0.012	0.051	0.22
Teacher reported:													
Child has poor hearing	4,658	0.890	0.805	0.984	0.02	0.901	0.748	1.086	0.27	0.881	0.728	1.068	0.20
Child has poor speech	4,729	0.792	0.732	0.856	<0.001	1.036	0.931	1.153	0.51	1.029	0.924	1.146	0.60
Child has poor eyesight	4,686	1.096	1.058	1.135	<0.001	1.137	1.033	1.252	0.009	1.135	1.029	1.251	0.01
Child is clumsy	4,703	0.856	0.813	0.901	<0.001	1.056	0.970	1.149	0.21	1.060	0.973	1.155	0.18
Parents interested in child’s education	4,529	1.175	1.147	1.204	<0.001	1.024	0.972	1.078	0.38	1.027	0.974	1.082	0.33
Parents expect child will stay in school	4,182	1.418	1.381	1.455	<0.001	1.032	0.969	1.098	0.33	1.033	0.970	1.101	0.31
Parents wishes child to get degree	4,176	1.356	1.322	1.391	<0.001	1.039	0.982	1.100	0.19	1.043	0.984	1.104	0.15
Child aspires to A-levels	4,261	1.337	1.306	1.370	<0.001	1.044	0.984	1.108	0.15	1.047	0.986	1.112	0.13
Child aspires to study full-time after leaving school	4,213	1.503	1.461	1.545	<0.001	1.076	1.018	1.136	0.010	1.078	1.020	1.139	0.008

^*^Mean differences reported. Robust standard errors. Adjusted analysis includes first twenty principal components of the genotype matrix.

**Table 4:** Association of number of O-levels and Rietveld allele score and educational attainment and log household income and wealth.

Education	N	Number of O-levels				Rietveld *et al*. (2013) allele score								
		Unadjusted				Unadjusted				Adjusted for population stratification				
			Confidence interval				Confidence Interval				Confidence interval			
		Coefficient	Lower	Upper	P-value	Coefficient	Lower	Upper	P-value	Coefficient	Lower	Upper	P-value	F-test
Number of O-levels*	5,458					0.070	0.003	0.137	0.04	0.072	0.005	0.139	0.03	4.46
Has A-levels$	5,458	1.987	1.918	2.059	<0.001	1.105	1.041	1.173	0.001	1.112	1.047	1.182	<0.001	11.62
Has degree$	5,458	1.467	1.424	1.513	<0.001	1.043	0.967	1.126	0.27	1.036	0.960	1.119	0.36	0.87
Income														
Log before-tax household income	4,353	0.074	0.065	0.083	<0.001	0.010	−0.012	0.033	0.37	0.010	−0.013	0.032	0.41	
Log net take home pay	3,979	0.107	0.095	0.119	<0.001	0.040	0.008	0.072	0.01	0.041	0.009	0.073	0.01	
Log family equivalised income	5,219	0.026	0.020	0.031	<0.001	0.008	−0.008	0.025	0.31	0.008	−0.008	0.025	0.33	
Log investments and savings wealth	4,387	0.211	0.180	0.242	<0.001	0.074	−0.009	0.157	0.08	0.075	−0.008	0.159	0.08	

^*^Mean differences reported.

^$^odds-ratios reported. Robust standard errors. Adjusted analysis includes first twenty principal components of the genotype matrix.

**Table 5:** Univariate estimates of chip-heritability for pre-natal phenotypes and their genetic correlation with number of O-levels.

	N	Genetic			Genetic correlation with number of O-levels		
		heritability ( 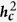 )	se	P-value	Corr (*r*_g_)	se	P-value*
Father’s age (years)	5,100	0.03	0.06	0.31	−0.95	1.31	0.11
Mother’s age (years)	5,255	0.03	0.06	0.31	−0.89	1.24	0.12
Mother’s weight (kg)	5,152	0.15	0.06	0.010	0.05	0.37	0.44
Father’s height (cm)	4,535	0.22	0.07	0.001	0.38	0.31	0.12
Mother’s height (cm)	5,054	0.12	0.07	0.03	0.14	0.41	0.37
Age father left school (years)	5,078	0.07	0.07	0.13	1.00	0.61	0.03
Age mother left school (years)	4,124	0.07	0.08	0.19	1.00	0.64	0.03
Average age parent’s left school (years)	3,874	0.02	0.08	0.39	1.00	1.12	0.50
Male	5,458	0.05	0.06	0.20	−0.80	0.74	0.09
Mother smoked	5,245	0.05	0.06	0.19	0.23	0.63	0.35
Mother smoked during pregnancy	5,197	0.10	0.06	0.04	0.02	0.43	0.48
Father high social class	5,023	0.23	0.07	<0.001	0.87	0.32	0.002
Paternal grandfather high social class	4,409	0.07	0.08	0.18	0.39	0.64	0.27
Maternal grandfather high social class	4,367	0.21	0.08	0.003	0.64	0.36	0.03

Estimated using GCTA. *P-value from one-sided likelihood ratio test of the hypothesis r_g_ = 0. All parameters adjusted for first twenty principle components of the genetic relatedness matrix and an indicator variable for the GWAS sample.

**Table 6:** Univariate estimates of chip-heritability for educational phenotypes at age 7 and 11 and genetic correlation with number of O-levels.

	N	Univariate			Genetic correlation with number of O-levels		
		heritability ( 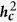 )	se	P-value	Corr (*r*_g_)	se	P-value*
Tests at age 7:							
Arithmetic	4,932	0.11	0.07	0.05	0.50	0.37	0.13
Reading	4,917	0.15	0.07	0.01	0.20	0.35	0.30
Teacher reported ability	4,951	0.17	0.07	0.007	1.00	0.31	0.002
Tests at age 11:							
Maths	4,770	0.23	0.07	<0.001	0.53	0.24	0.05
Reading	4,771	0.28	0.07	<0.001	0.61	0.22	0.02
Verbal	4,773	0.11	0.07	0.06	0.64	0.35	0.09
Nonverbal	4,773	0.06	0.07	0.20	0.94	0.53	0.07
Copying	4,760	0.12	0.07	0.04	0.14	0.41	0.37
Teacher reported ability	4,719	0.13	0.07	0.03	0.71	0.30	0.05
Child’s intentions and expectations:							
Parents interested in child’s education	4,693	0.02	0.07	0.37	0.81	1.02	0.16
Intends to stay on past min. age	4,683	0.18	0.07	0.007	0.78	0.37	0.02

Estimated using GCTA. *P-value from one-sided likelihood ratio test of the hypothesis *r*_g_ = 0. All parameters adjusted for first twenty principle components of the genetic relatedness matrix and an indicator variable for the GWAS sample.

**Table 7:** Univariate estimates of chip-heritability for educational phenotypes at age 16 and genetic correlation with number of O-levels.

	N	Univariate			Genetic correlation with number of O-levels		
		heritability ( 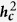 )	se	P-value	Corr (*r*_g_)	se	P-value*
Tests							
Maths	4,235	0.17	0.08	0.02	0.59	0.25	0.06
Reading	4,253	0.26	0.08	<0.001	0.53	0.24	0.05
Teacher reported ability							
Maths	4,139	0.00	0.08	0.50	−1.00	2.03	0.50
English	4,223	0.20	0.08	0.005	0.45	0.34	0.11
Science	3,543	0.00	0.09	0.49	1.00	2.24	0.22
Lazy 1-2-3-4-5 Hardworking	4,308	0.21	0.08	0.003	0.48	0.31	0.09
Teacher reported:							
Child has poor hearing	4,658	0.06	0.07	0.22	0.72	0.78	0.12
Child has poor speech	4,729	0.00	0.07	0.50	1.00	24.79	0.40
Child has poor eyesight	4,686	0.01	0.07	0.42	0.58	1.79	0.32
Child is clumsy	4,703	0.05	0.07	0.24	−0.26	0.66	0.35
Parents interested in child’s education	4,529	0.08	0.07	0.13	0.49	0.59	0.21
Parents wishes child would get a degree	4,182	0.12	0.08	0.05	0.76	0.39	0.06
Parents expect child will stay in school	4,176	0.05	0.08	0.28	1.00	0.91	0.12
Child aspires to A-levels	4,261	0.15	0.08	0.03	1.00	0.41	0.01
Child aspires to study full-time after leaving school	4,213	0.19	0.08	0.01	0.62	0.28	0.05

Estimated using GCTA. *P-value from one-sided likelihood ratio test of the hypothesis *r*_g_ = 0. All parameters adjusted for first twenty principle components of the genetic relatedness matrix and an indicator variable for the GWAS sample.

**Table 8:** Univariate estimates of chip-heritability for educational and income phenotypes and genetic correlation with number of O-levels.

	N	Univariate			Genetic correlation with number of O-levels		
		heritability ( 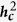 )	se	P- value	Corr (*r*_g_)	se	P- value*
Education							
Number of O-levels	5,458	0.10	0.06	0.05			
Has A-levels	5,458	0.11	0.06	0.03	0.78	0.23	0.04
Has degree	5,458	0.13	0.06	0.01	0.96	0.35	0.007
Income							
Log before-tax household income	4,353	0.00	0.08	0.50	−1.00	3.71	0.50
Log net take home pay	3,979	0.13	0.08	0.05	0.11	0.44	0.41
Log family equivalised income	5,219	0.06	0.06	0.19	1.00	0.80	0.05
Log investments and savings wealth	4,387	0.10	0.08	0.09	−0.04	0.50	0.47

Notes: Estimated using GCTA. *P-value from one-sided likelihood ratio test of the hypothesis *r*_g_ = 0. All parameters adjusted for first twenty principle components of the genetic relatedness matrix and an indicator variable for the GWAS sample.
