# Supplementary material for: The role of common genetic variation in educational attainment and income: evidence from the National Child Development Study
Source: Sci Rep. 2015 Nov 12;5:16509. doi: 10.1038/srep16509 (PMC4642349; doi:10.1038/srep16509)
Supplement: Supplementary Materials [file srep16509-s1.pdf]

The role of common genetic variation in educational attainment and income: evidence from the National Child Development Study

Neil M Davies*[1,2], Gibran Hemani[1,2], Nic J Timpson[1,2], Frank Windmeijer,[1,3] and George Davey Smith[1,2]

[1] Medical Research Council Integrative Epidemiology Unit, University of Bristol, BS8 2BN, United Kingdom.

[2] School of Social and Community Medicine, University of Bristol, Barley House, Oakfield Grove, Bristol, BS8 2BN, United Kingdom.

[3] Department of Economics, University of Bristol, 8 Woodland Road, Bristol BS8 1TN, United Kingdom.

Supplementary Figure 1: Flow chart of participants' inclusion in the study

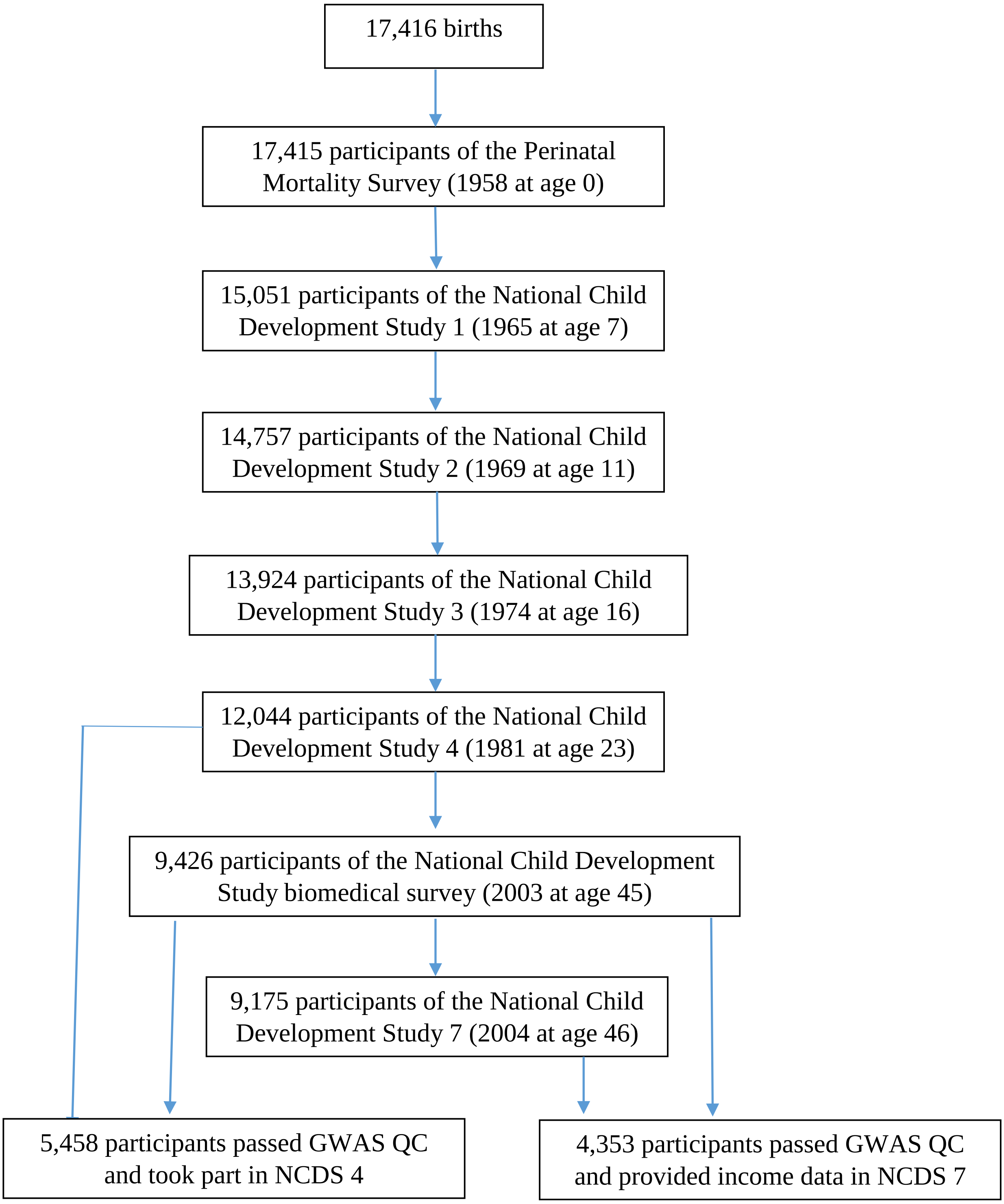

Supplementary Figure 2: The association of the Rietveld *et al.* (2013) allele score and likelihood of achieving A-levels by age 23.

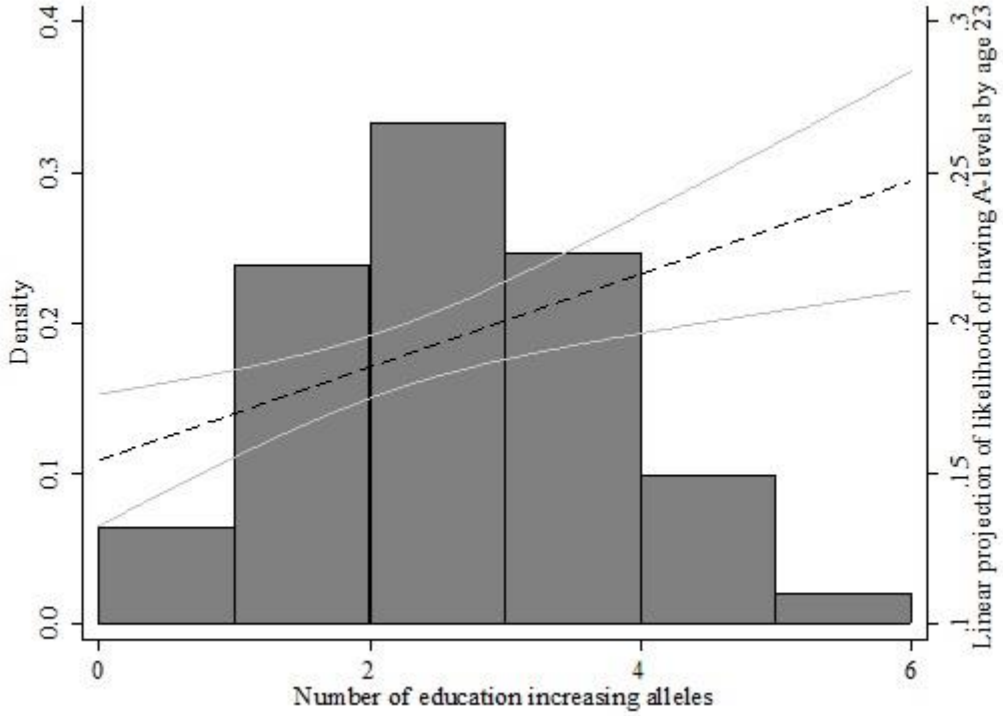

Notes: The dashed line indicates linear prediction of household income based on the number of alleles. The grey line indicates the confidence intervals of linear prediction.

Supplementary Figure 3: The association of the Rietveld et al. (2013) allele score and household income at age 46.

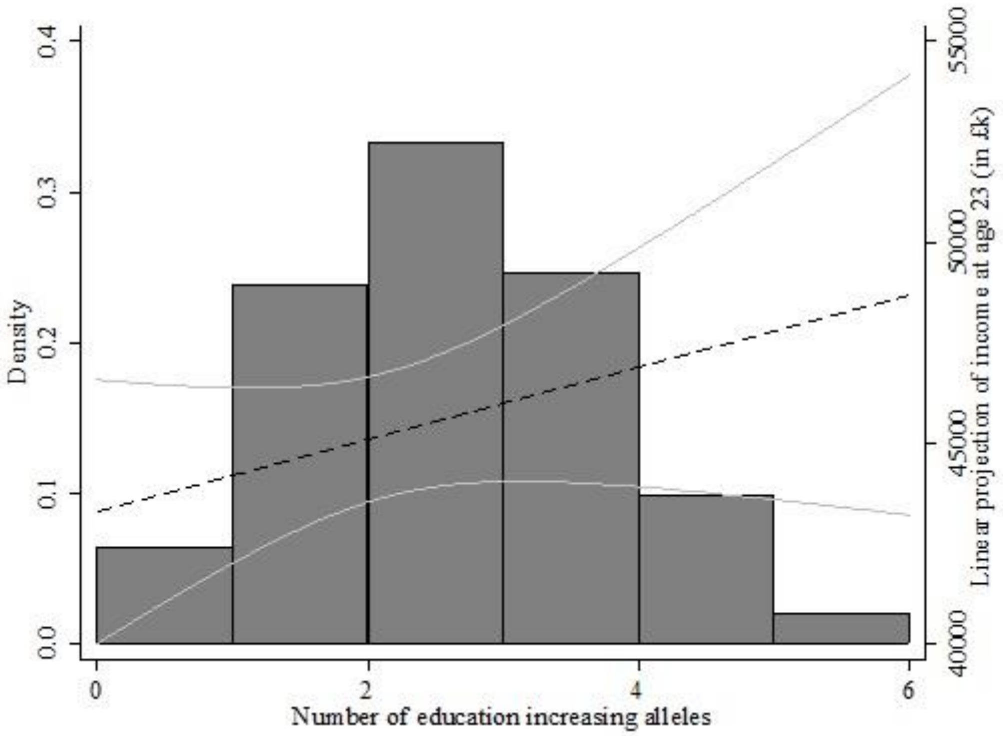

Notes: The dashed line indicates linear prediction of household income based on the number of alleles. The grey line indicates the confidence intervals of linear prediction.

Supplementary Figure 4: Diagram illustrating possible sources of bivariate chip-heritability.

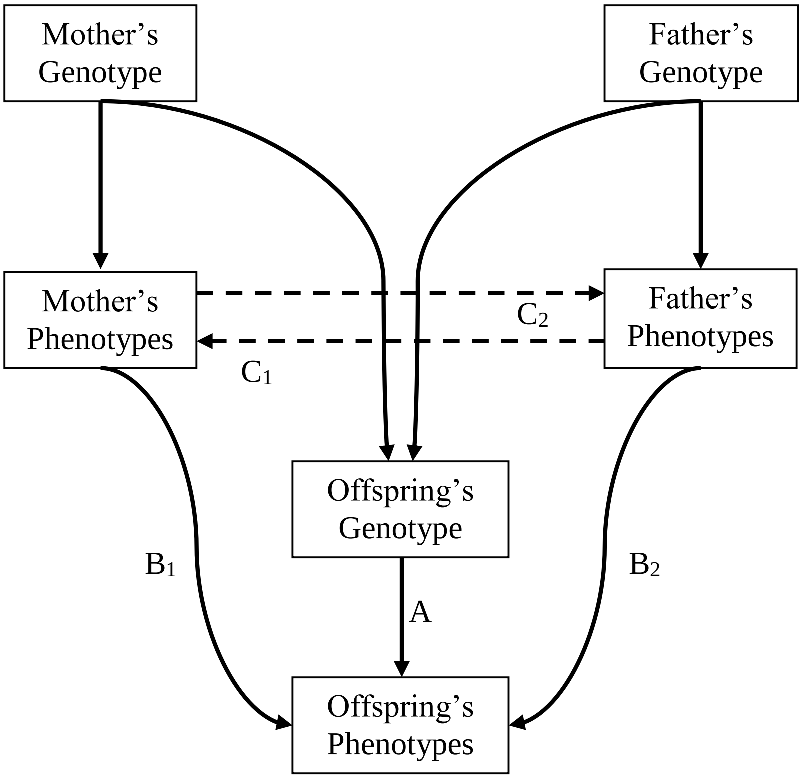

Notes: Arrow A represents the direct effect of the offspring's genotype on the phenotypes, B_1 and B_2 represent the dynastic (direct) effect of the parents' phenotypes on the offspring's outcomes, and C_1 and C_2 are the association of parents' phenotypes due to assortative mating and unmeasured population stratification. Our estimates of bivariate chip-heritability may overestimate A, because of the contribution of dynastic effects, assortative mating and population stratification.

Supplementary Table 1: Descriptive statistics of 5,458 participants of the National Child Development Study included in the analysis.

	N	Mean	Standard deviation	Minimum	Maximum
**From the survey at birth (1958):**					
Father's age (years)	5,100	30.5	6.2	16.0	57.0
Mother's age (years)	5,255	27.4	5.5	15.0	46.0
Mother's weight (kg)	5,152	59.6	9.5	41.3	98.4
Father’s height (cm)	4,535	174.7	7.4	142.2	200.7
Mother's height (cm)	5,054	161.3	6.3	127.0	182.9
Age father left school (years)	5,078	14.8	2.1	14.0	31.0
Age mother left school (years)	4,124	15.5	1.6	12.5	23.0
Average age parents left school (years)	3,874	15.2	1.6	13.3	26.5
Male	5,458	0.51		0.00	1.00
Mother smoked	5,245	0.41		0.00	1.00
Mother smoked during pregnancy	5,197	0.33		0.00	1.00
Father high social class	4,720	0.25		0.00	1.00
Paternal grandfather high social class	5,023	0.19		0.00	1.00
Maternal grandfather high social class	4,409	0.15		0.00	1.00

Supplementary Table 2: Descriptive statistics of 5,458 participants of the National Child Development Study included in the analysis.

From the survey at age 7 (1965):	N	Mean	Standard deviation	Minimum	Maximum
Arithmetic	4,932	5.3	2.4	0.0	10.0
Reading	4,917	3.7	1.3	0.0	5.0
Teacher reported ability	4,951	10.3	3.5	0.0	20.0
**From the survey at age 11 (1969):**					
Maths tests score	4,770	18.0	10.2	0.0	40.0
Reading tests score	4,771	16.7	5.9	0.0	35.0
Verbal tests score	4,773	23.4	8.9	0.0	40.0
Nonverbal tests score	4,773	22.0	7.2	0.0	40.0
Copying tests score	4,760	8.5	1.4	0.0	12.0
Teacher reported ability	4,719	8.3	2.9	0.0	16.0
Parents interested in child's education	4,693	0.60		0.00	1.00
Intends to stay on past min. age	4,683	0.29		0.00	1.00
**From the survey at age 16 (1974):**					
Maths test score	4,235	13.7	7.0	0.0	31.0
Reading test score	4,253	26.3	6.3	1.0	35.0
Teacher reported ability					
Maths	4,139	0.18		0.00	1.00
English	4,223	0.24		0.00	1.00
Science	3,543	0.18		0.00	1.00
Lazy 1-2-3-4-5 Hardworking	4,308	3.3		1.0	5.0
Teacher reported:					
Child has poor hearing	4,658	0.02		0.00	1.00
Child has poor speech	4,729	0.05		0.00	1.00
Child has poor eyesight	4,686	0.07		0.00	1.00
Child is clumsy	4,703	0.09		0.00	1.00
Parents interested in child's education					
Parents expect child will stay in school	4,529	0.61		0.00	1.00
Parents wishes child will get degree	4,182	0.25		0.00	1.00
Child aspires to A-levels	4,176	0.34		0.00	1.00
Child aspires to study full-time after leaving school	4,261	0.28		0.00	1.00
**From the survey at age 23 (1981):**					
Number of O-levels	5458	2.0	2.8	0.0	9.0
Has A-levels	5,458	0.19		0.00	1.00
Has degree	5,458	0.11		0.00	1.00
Equivalent net weekly family income (£)	5,219	119	58	2	816
**From the survey at age 46 (2004):**					
Household income net of tax (£s)	4,353	45,247	52,676	1	999,999
**From the survey at age 50 (2008):**					
Net take home pay (£)	3,979	1,669	7,620	1	323,000
Savings and investment wealth (£k)	4,387	68	849	0	50,000

Supplementary Table 3: Frequency of rs9320913, rs11584700, and rs4851266 in the National Child Development Study.

rs ID	Chr.	Pos.	Alleles: More educated	Alleles: Less educated	Effect allele frequency	Hardy Weinberg Test: χ^2	Hardy Weinberg Test: p-value	Rietveld Weight[1]
rs11584700	1	204607855	G	A	0.21	0.54	0.46	0.095
rs4851266	2	100202017	T	C	0.39	1.42	0.23	0.082
rs9320913	6	98136857	A	C	0.47	0.02	0.89	0.101

Notes: Position indicated on GRCh38 38.1/141. Rietveld et al. (2013) weights taken from Tables S8 and S9 and are from the GWAS on number of years of education (EduYears).

Supplementary Table 4: Difference in association of allele score and educational attainment and household income in children of high versus low socioeconomic position fathers.

		Unadjusted				Adjusted for population stratification			
	N	Coefficient	Lower CI	Upper CI	P-value	Coefficient	Lower CI	Upper CI	P-value
Number of O-levels*									
allele score	5,023	0.033	-0.037	0.103	0.36	0.039	-0.031	0.108	0.27
interaction		0.198	-0.006	0.403	0.06	0.188	-0.014	0.390	0.07
A-levels$									
allele score	5,023	1.109	1.025	1.199	0.010	1.121	1.035	1.213	0.005
interaction		1.023	0.888	1.179	0.75	1.016	0.881	1.173	0.82
Degree$									
allele score	5,023	1.050	0.942	1.171	0.38	1.047	0.938	1.168	0.42
interaction		0.996	0.840	1.182	0.96	0.991	0.834	1.177	0.92
Income (£k)*									
allele score	4,005	1,274	189	2,359	0.02	1,339	242	2,435	0.02
interaction		-1,994	-5,581	1,593	0.28	-2,169	-5,762	1,425	0.24

Notes: Robust standard errors, * odds-ratios reported, $ mean differences reported. Interactions indicate the association of the allele score outcome in children of high socioeconomic position fathers. Adjusted analysis includes first twenty principal components of the genotype matrix.

Supplementary Table 5: Association of number of O-levels and Rietveld *et al.* (2013) genome-wide allele score and perinatal covariates.

		Number of O-levels				Rietveld et al. (2013) genome-wide allele score							
		Unadjusted				Unadjusted				Adjusted for population stratification			
Outcome:	N	Mean difference	Lower CI	Upper CI	P-value	Mean difference	Lower CI	Upper CI	P-value	Mean difference	Lower CI	Upper CI	P-value
Father's age (years)	5,100	0.149	0.088	0.209	<0.001	0.204	0.030	0.378	0.02	0.191	0.013	0.368	0.04
Mother's age (years)	5,255	0.145	0.092	0.197	<0.001	0.270	0.121	0.419	<0.001	0.272	0.119	0.424	<0.001
Mother's weight (kg)	5,152	-0.017	-0.104	0.069	0.69	-0.086	-0.351	0.179	0.53	-0.071	-0.344	0.202	0.61
Father’s height (cm)	4,535	0.226	0.166	0.286	<0.001	0.395	0.177	0.613	<0.001	0.461	0.239	0.683	<0.001
Mother's height (cm)	5,054	0.349	0.278	0.420	<0.001	0.041	-0.138	0.220	0.66	0.124	-0.058	0.306	0.18
Age father left school (years)	5,078	0.215	0.188	0.242	<0.001	0.267	0.204	0.330	<0.001	0.277	0.214	0.341	<0.001
Age mother left school (years)	4,124	0.175	0.152	0.197	<0.001	0.166	0.118	0.215	<0.001	0.174	0.124	0.224	<0.001
Average age parent’s left school (years)	3,874	0.205	0.181	0.229	<0.001	0.219	0.167	0.271	<0.001	0.229	0.176	0.281	<0.001
Male*	5,458	0.987	0.968	1.005	0.15	1.016	0.962	1.072	0.57	1.013	0.958	1.070	0.65
Mother smoked*	5,245	0.915	0.897	0.934	<0.001	0.939	0.888	0.994	0.03	0.915	0.863	0.970	0.003
Mother smoked during pregnancy*	5,197	0.902	0.882	0.922	<0.001	0.927	0.873	0.983	0.01	0.901	0.848	0.959	<0.001
Father high social class*	5,023	1.251	1.222	1.280	<0.001	1.361	1.264	1.466	<0.001	1.385	1.283	1.495	<0.001
Paternal grandfather high social class*	4,409	1.129	1.100	1.159	<0.001	1.261	1.157	1.374	<0.001	1.267	1.161	1.384	<0.001
Maternal grandfather high social class*	4,367	1.156	1.129	1.184	<0.001	1.301	1.202	1.409	<0.001	1.309	1.206	1.421	<0.001

Notes: * Odds-ratios reported. Robust standard errors. Adjusted analysis includes first twenty principal components of the genotype matrix. Genome-wide allele score normalized to mean zero standard deviation one.

Supplementary Table 6: Association of number of O-levels and Rietveld genome-wide allele score and educational phenotypes at age 7.

		Number of O-levels				Rietveld et al. (2013) genome-wide allele score							
		Unadjusted				Unadjusted				Adjusted for population stratification			
Outcome:	N	Mean difference	Lower CI	Upper CI	P-value	Mean difference	Lower CI	Upper CI	P-value	Mean difference	Lower CI	Upper CI	P-value
Tests:													
Arithmetic	4,932	0.301	0.279	0.323	<0.001	0.324	0.256	0.392	<0.001	0.328	0.258	0.397	<0.001
Reading	4,917	0.146	0.135	0.156	<0.001	0.195	0.160	0.230	<0.001	0.198	0.162	0.234	<0.001
Teacher reported ability	4,951	0.561	0.529	0.593	<0.001	0.581	0.483	0.680	<0.001	0.601	0.500	0.702	<0.001

Notes: Robust standard errors. Adjusted analysis includes first twenty principal components of the genotype matrix. Genome-wide allele score normalized to mean zero standard deviation one.

Supplementary Table 7: Association of number of O-levels and Rietveld genome-wide allele score and educational phenotypes at age 11.

		Number of O-levels				Rietveld et al. (2013) genome-wide allele score							
		Unadjusted				Unadjusted				Adjusted for population stratification			
Outcome:	N	Mean difference	Lower CI	Upper CI	P-value	Mean difference	Lower CI	Upper CI	P-value	Mean difference	Lower CI	Upper CI	P-value
Test scores:													
Maths	4,770	1.901	1.817	1.985	<0.001	2.098	1.808	2.387	<0.001	2.180	1.885	2.476	<0.001
Reading	4,771	1.061	1.010	1.112	<0.001	1.131	0.959	1.304	<0.001	1.191	1.014	1.368	<0.001
Verbal	4,773	1.451	1.378	1.524	<0.001	1.658	1.401	1.915	<0.001	1.726	1.463	1.989	<0.001
Nonverbal	4,773	1.147	1.086	1.208	<0.001	1.164	0.958	1.371	<0.001	1.222	1.011	1.434	<0.001
Copying	4,760	0.106	0.091	0.120	<0.001	0.082	0.040	0.123	<0.001	0.101	0.058	0.143	<0.001
Teacher reported ability	4,719	0.529	0.504	0.555	<0.001	0.595	0.512	0.678	<0.001	0.620	0.535	0.705	<0.001
Child's intentions and expectations													
Parents interested in child's education*	4,693	1.166	1.139	1.194	<0.001	1.120	1.054	1.190	<0.001	1.130	1.062	1.202	<0.001
Intends to stay on past min. age*	4,683	1.163	1.138	1.188	<0.001	1.154	1.082	1.231	<0.001	1.154	1.080	1.233	<0.001

Notes: * Odds-ratios reported. Robust standard errors. Adjusted analysis includes first twenty principal components of the genotype matrix. Genome-wide allele score normalized to mean zero standard deviation one.

Supplementary Table 8: Association of number of O-levels and Rietveld genome-wide allele score and educational phenotypes at age 16.

		Number of O-levels				Rietveld et al. (2013) genome-wide allele score							
		Unadjusted				Unadjusted				Adjusted for population stratification			
Outcome:	N	Odds-ratio	Lower CI	Upper CI	P-value	Odds-ratio	Lower CI	Upper CI	P-value	Odds-ratio	Lower CI	Upper CI	P-value
Tests:													
Maths*	4,235	1.464	1.405	1.522	<0.001	1.370	1.159	1.580	<0.001	1.445	1.229	1.661	<0.001
Reading*	4,253	1.080	1.032	1.128	<0.001	1.098	0.910	1.286	<0.001	1.162	0.969	1.354	<0.001
Teacher reported ability													
Maths	4,139	1.263	1.231	1.295	<0.001	1.237	1.140	1.342	<0.001	1.234	1.135	1.343	<0.001
English	4,223	1.165	1.139	1.192	<0.001	1.250	1.163	1.344	<0.001	1.262	1.171	1.360	<0.001
Science	3,543	1.214	1.181	1.248	<0.001	1.270	1.160	1.391	<0.001	1.269	1.156	1.391	<0.001
Lazy 1-2-3-4-5 Hardworking*	4,308	0.151	0.139	0.162	<0.001	0.122	0.086	0.158	<0.001	0.137	0.101	0.174	<0.001
Teacher reported:													
Child has poor hearing	4,658	0.890	0.805	0.984	0.02	0.984	0.820	1.180	0.86	0.955	0.789	1.156	0.64
Child has poor speech	4,729	0.792	0.732	0.856	<0.001	0.970	0.858	1.096	0.63	0.940	0.831	1.064	0.33
Child has poor eyesight	4,686	1.096	1.058	1.135	<0.001	1.109	0.993	1.239	0.07	1.106	0.986	1.240	0.08
Child is clumsy	4,703	0.856	0.813	0.901	<0.001	0.909	0.819	1.008	0.07	0.890	0.800	0.990	0.03
Parents interested in child's education	4,529	1.175	1.147	1.204	<0.001	1.118	1.053	1.188	<0.001	1.141	1.072	1.215	<0.001
Parents expect child will stay in school	4,182	1.418	1.381	1.455	<0.001	1.433	1.330	1.545	<0.001	1.452	1.345	1.568	<0.001
Parents wishes child to get degree	4,176	1.356	1.322	1.391	<0.001	1.349	1.261	1.443	<0.001	1.354	1.263	1.451	<0.001
Child aspires to A-levels	4,213	1.503	1.461	1.545	<0.001	1.413	1.322	1.511	<0.001	1.432	1.337	1.534	<0.001
Child aspires to study full-time after leaving school	4,261	1.337	1.306	1.370	<0.001	1.354	1.260	1.455	<0.001	1.367	1.270	1.472	<0.001

Notes: * Mean differences reported. Robust standard errors. Adjusted analysis includes first twenty principal components of the genotype matrix. Genome-wide allele score normalized to mean zero standard deviation one.

Supplementary Table 9: Association of number of O-levels and Rietveld genome-wide allele score and educational phenotypes at age 23 and household income at age 46.

		Number of O-levels				Rietveld et al. (2013) genome-wide allele score								
		Unadjusted				Unadjusted				Adjusted for population stratification				
Outcome:	N	Coefficient	Lower CI	Upper CI	P-value	Coefficient	Lower CI	Upper CI	P-value	Coefficient	Lower CI	Upper CI	P-value	F-test
Number of O-levels*	5,458					0.433	0.355	0.512	<0.001	0.477	0.398	0.555	<0.001	140.07
Has A-levels$	5,458	1.987	1.918	2.059	<0.001	1.440	1.340	1.547	<0.001	1.491	1.383	1.607	<0.001	112.95
Has degree$	5,458	1.467	1.424	1.513	<0.001	1.484	1.351	1.630	<0.001	1.493	1.357	1.642	<0.001	66.12

Notes: * Mean differences reported, $ odds-ratios reported. Robust standard errors. Adjusted analysis includes first twenty principal components of the genotype matrix. Cragg-Donald F-statistic reported. Genome-wide allele score normalized to mean zero standard deviation one.

Supplementary Table 10: Association of number of O-levels and Rietveld genome-wide allele score and log income and wealth at age 46.

		Number of O-levels				Rietveld et al. (2013) genome-wide allele score							
		Unadjusted				Unadjusted				Unadjusted			
	N	Coef	Lower CI	Upper CI	P-value	Coef	Lower CI	Upper CI	P-value	Coef	Lower CI	Upper CI	P-value
Log household income before tax	4,353	0.074	0.065	0.083	<0.001	0.061	0.034	0.088	<0.001	0.062	0.035	0.089	<0.001
Log net take home pay	3,979	0.107	0.095	0.119	<0.001	0.138	0.101	0.175	<0.001	0.138	0.100	0.176	<0.001
Log family equivalised income	5,219	0.026	0.020	0.031	<0.001	0.040	0.022	0.058	<0.001	0.043	0.024	0.061	<0.001
Log investments and savings wealth	4,387	0.211	0.180	0.242	<0.001	0.296	0.202	0.391	<0.001	0.323	0.225	0.421	<0.001

Notes: Genome-wide allele score normalized to mean zero standard deviation one.
